# Stigmergy: from mathematical modelling to control

**DOI:** 10.1098/rsos.240845

**Published:** 2024-09-04

**Authors:** Alain Boldini, Martina Civitella, Maurizio Porfiri

**Affiliations:** ^1^ Department of Mechanical and Aerospace Engineering, New York University Tandon School of Engineering, Brooklyn, NY 11201, USA; ^2^ Center for Urban Science and Progress, New York University Tandon School of Engineering, Brooklyn, NY 11201, USA; ^3^ Department of Mechanical Engineering, New York Institute of Technology, Old Westbury, NY 11568, USA; ^4^ Department of Biomedical Engineering, New York University Tandon School of Engineering, Brooklyn, NY 11201, USA

**Keywords:** animal behaviour, collective behaviour, control, swarms

## Abstract

Stigmergy, the indirect communication between agents of a swarm through dynamic environmental modifications, is a fundamental self-organization mechanism of animal swarms. Engineers have drawn inspiration from stigmergy to establish strategies for the coordination of swarms of robots and of mixed societies of robots and animals. Currently, all models of stigmergy are algorithmic, in the form of behavioural rules implemented at an individual level. A critical challenge for the understanding of stigmergic behaviour and translation of stigmergy to engineering is the lack of a holistic approach to determine which modifications of the environment are necessary to achieve desired behaviours for the swarm. Here, we propose a mathematical framework that rigorously describes the relationship between environmental modifications and swarm behaviour. Building on recent strides in continuification techniques, we model the swarm and environmental modifications as continua. This approach allows us to design the environmental modifications required for the swarm to behave as desired. Through analytical derivations and numerical simulations of one- and two-dimensional examples, we show that our framework yields the distribution of traces required to achieve a desired formation. Such an approach provides an adaptable framework for different implementation platforms, from robotic swarms to mixed societies of robots and animals.

## 1. Introduction

Stigmergy—defined as a ‘mechanism of indirect coordination in which the trace left by an action in a medium stimulates subsequent actions’ [1]—is a fundamental means of self-organization in complex systems [2]. The concept of stigmergy originated from the study of coordination in groups of animals [3]. For example, ants release pheromones in the environment [4] to guide other ants back to the nest once they find food [5], or to cooperatively transport large food items [6]. Likewise, pumas and other large Felidae leave traces in the environment to mark territory so that other conspecifics would avoid the area [7]. In animal colonies that build structures [8,9], such as wasps [10] and termites [11,12], stigmergy allows the incredible coordination of thousands of individuals to build intricate nests, without any pre-planning and central coordination.

With the advent of robotic systems, engineers have taken inspiration from stigmergy in natural swarms to build self-organizing groups of robots that coordinate to achieve a desired goal [13–15]. In stigmergy, robots in a swarm not only coordinate to sense the environment [16,17], but they actively modify it to communicate with each other. Through stigmergy, robotic swarms can gather objects [18], sort them [15], navigate unknown environments [19], and search and track a moving target [20]. Stigmergy is particularly promising for collective construction, as the structure that is being built can be utilized as the stigmergic signal itself [21,22]. In this vein, extensions of stigmergic approaches have been proposed to achieve precise and accurate collective construction [23,24]. Stigmergy is an important coordination strategy not only in biological and robotic systems but also in so-called ‘mixed societies’ that integrate biological and robotic individuals [25,26]. Therein, robots are used to create new collective responses or to elicit a desired behaviour from the animal group [27]. In addition to direct social interactions between animals and robots, stigmergic stimuli released by robots have been often used to achieve the desired swarm behaviour, for example through light signals [28] or pheromone release [29] by robots.

A fundamental challenge for biologists is to reconstruct how environmental modifications implemented at an individual level, without a central coordination, trigger a complex behaviour of the swarm, such as the construction of a nest or food gathering. The literature relies on agent-based modelling grounded in individual behavioural rules [13–15], which do not allow a holistic analysis of the swarm. Currently, no stigmergic model allows the study of the overall distribution of environmental modifications that enable these complex behaviours. Similarly, engineers are faced with the design problem of how to control robotic agents toward achieving a desired behaviour for the swarm, be it robotic or mixed. Such a challenge requires the development of a mathematically tractable and interpretable framework for stigmergy that could complement existing algorithmic implementations, which rely on a sequence of discrete behavioural rules implemented at the individual level.

**Figure 1.**
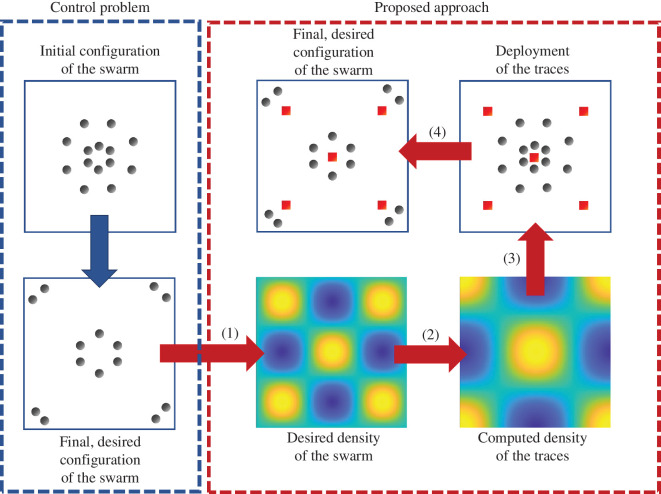
Schematics of the control problem and proposed framework for our mathematical model of stigmergy. The control problem that we seek to address is achieving a desired configuration of a large swarm in terms of position of its discrete units. Such a desired configuration is continuified (1) to provide the desired density of the swarm. The density of the traces is computed (2) based on the desired density of the swarm, and is then discretized (3) to allow actual deployment of the traces. The final, desired configuration of the swarm arises from the interaction between units of the swarm and the traces left (4).

Here, we put forward a control-oriented mathematical backdrop to holistically describe environmental modifications in animal swarms and engineer stigmergic interactions in robotic and mixed swarms, where units of the swarm interact with each other and with dynamic modifications of the environment that we identify as ‘traces’. Our mathematical framework is based on recent advancements in the field of control of large multi-agent systems, which relies on a ‘continuification’ of the equations of motion of the swarm [30,31] (see figure 1). In this vein, rather than modelling and controlling individual motions [32], we model the swarm as a continuous fluid and describe the spatio-temporal evolution of its density, similar to thermodynamic approaches for large-scale systems [33] and former robot density control algorithms [34–38]. Our work differs from previous studies on continuification-based control of large swarms [30,31] as we do not apply a control action to each swarm unit. In animal swarms and mixed societies, it is not possible to directly control biological units, and even in robotic swarms controlling each unit would require a centralized approach or, at least, some form of connectivity in the swarm [39]. Based on these groundings, we focus on the realistic case where the control engineer can only design dynamic modifications of the environment, such that no centralization or communication between the units is needed.

While our work shares the same backdrop of continuified swarms as that in Maffettone *et al*. [30], it tackles the control problem through a totally different perspective. Our control action is not directly applied to the swarm agents; rather, it is mediated by the deposition of traces in the environment. Such a continuified view of stigmergy is an original contribution of this paper, which allows us for the first time to explicitly compute the trace distribution necessary to achieve a desired swarm configuration. In this vein, we offer closed-form solutions of the control input in the form of density of traces.

We examine a prototypical example of a task that can be achieved through stigmergy: shape formation [21,40]. We seek to compute the distribution of traces that make the swarm achieve a desired formation, that is, a prescribed density profile. To this end, we propose a general procedure to compute the density of traces that allows us to retrieve the prescribed density of the swarm. Once the density of traces is known, it is discretized to allow for deployment in a real environment, in a discrete form. We do not focus on implementation details of how such density profile of traces can be generated, as we concentrate on the mathematical modelling of the overarching stigmergic control strategy, generalizable to a variety of practical implementations. While one could describe the dynamics of deposition of the traces, such a process would be heavily context-dependent (for example, the dynamics of ants’ pheromone release [13] is different from that of robotic collective additive manufacturing (AM) [23]), thus hindering the generality of our theory.

We demonstrate the potential of our approach through a series of one- (1D) and two-dimensional (2D) case studies. In the 1D benchmarks, we show that our approach allows for the swarm to achieve a stationary formation and generate travelling waves [41]. The 2D simulations validate our approach in a more realistic scenario. We select a historical, fascinating example, by replicating the complex, non-uniform motion of the robotic lion of Leonardo da Vinci along a wavy circle [42] with our swarm.

## 2. Mathematical model

We start by introducing the general framework of our problem in a 1D setting, considering a swarm coordinating over a ring (§2.1). We define two types of interactions between units on the ring (§2.2): a purely repulsive interaction and an attractive–repulsive interaction. In §2.3, we put forward analytical solutions for both of these types of interactions. We then introduce a numerical method to study the time-evolution of the swarm at the continuum level in the 1D problem (§2.4), along with the discrete counterpart used to validate our approach (§2.5). Finally, in §2.6, we extend our mathematical model to multi-dimensional problems.

### 2.1. General framework in one dimension

Our aim is that of controlling a swarm moving on a ring ( $S^{1}$ ) [43], parametrized with the angle x∈[−π,π) . The state of the swarm is described by the density $\rho :{𝕊}^{1}\times {\mathbb{R}}_{+}\to {\mathbb{R}}_{+}$ . We assume that the evolution in time of the density is governed by mass conservation:


(2.1) $$\rho_{t}+(\rho v{)}_{x}=0,$$

where $(\cdot {)}_{t}$ and $(\cdot {)}_{x}$ indicate partial derivatives in time and space. Here, $v:{𝕊}^{1}\times {\mathbb{R}}_{+}\to \mathbb{R}$ is the velocity of the swarm.

We adopt a kinematic model [44] that neglects inertia effects, such that the velocity v depends explicitly on the density through a circular convolution [45]:


(2.2) $$v(x,t)=\int_{-\pi }^{\pi }q_{T}\;(x-y)\rho (y,t)\;dy+f(x,t),$$

where $q_{T}:{𝕊}^{1}\to \mathbb{R}$ is an interaction kernel and $f:{𝕊}^{1}\times {\mathbb{R}}_{+}\to \mathbb{R}$ is an external forcing term. The circular convolution is equivalent to the linear convolution over ℝ of a periodic function defined over ${𝕊}^{1}$ with a non-periodic kernel defined over ℝ (see §2.2). The kernel embodies the interactions between swarm units, typically derived from a potential, which may be repulsive, attractive or a combination of the two modulated by the distance [43]. By construction, the circular convolution in equation (2.2) guarantees that $\lim_{x\to \pi }\;v(x,t)=v(-\pi ,t)$ , so that $\rho (-\pi ,t)v(-\pi ,t)=\lim_{x\to \pi }\;\rho (x,t)v(x,t)$ . Thus, the total mass of the swarm $\int_{-\pi }^{\pi }\;\rho (y,t)\;dy=M$ is conserved. The model in equations (2.1) and (2.2) naturally arises from the continuification of classical, discrete kinematic models that have been widely used to describe agents’ behaviour in swarms [43].

We seek to develop a control strategy where the external forcing is provided by some ‘traces’ with density $\rho_{\mathrm{tr}}:{𝕊}^{1}\times {\mathbb{R}}_{+}\to \mathbb{R}$ , interacting with the swarm, such that


(2.3) $$f(x,t)=\int_{-\pi }^{\pi }\;q_{T}(x-y)\rho_{tr}\;(y,t)\;dy.$$

Since f is defined as a circular convolution, $\lim_{x\to \pi }\;f(x,t)=f(-\pi ,t)$ by design. Here and in the following, we adopt the same interaction kernel $q_{T}$ for the interaction between units in the swarm and the traces. Should the units of the swarm be able to distinguish between their peers and the traces, different interaction kernels can be utilized. We accept the possibility that $\rho_{\mathrm{tr}}$ may be negative as a computational device to simplify our derivations; we can recover a non-negative $\rho_{\mathrm{tr}}$ with equivalent effects, as made clear in §2.3.

### 2.2. Periodicization of the interaction kernels

Periodic interaction kernels $q_{T}$ are obtained from the periodicization of standard non-periodic kernels q :


(2.4) $$q_{T}(x)=\sum_{k=-\infty }^{\infty }q(x+2k\pi ).$$

We consider the following two standard non-periodic kernels.

**Repulsive kernel.** The non-periodic repulsive kernel is in the form


(2.5) $$q(x)=sgn(x)e^{-\frac{\left|x\right|}{L}},$$

where sgn is the sign function. Note that we utilize a length-scale L while fixing the domain to [−π,π) . Periodicization leads to


(2.6) $$q_{T}(x)=\sum_{k=-\infty }^{\infty }sgn(x+2k\pi )\;e^{-\frac{\left|x+2k\pi \right|}{L}}.$$

By separating the infinite series into two other infinite series based on the sign of x+2kπ and computing each of these series individually leads to


(2.7) $$q_{T}(x)=\frac{1}{e^{\frac{2\pi }{L}}-1}sgn(x)\left[e^{\frac{2\pi -|x|}{L}}-e^{\frac{\left|x\right|}{L}}\right].$$

**Morse kernel.** The non-periodic Morse kernel is an attractive–repulsive kernel, in the form


(2.8) $$q(x)=sgn(x)\left[\frac{1}{L_{R}}e^{-\frac{\left|x\right|}{L_{R}}}-\frac{\alpha }{L_{A}}e^{-\frac{\left|x\right|}{L_{A}}}\right]=\frac{1}{L_{R}}q_{R}(x)-\frac{\alpha }{L_{A}}q_{A}(x),$$

having defined


(2.9) $$q_{i}(x)=sgn(x)e^{-\frac{\left|x\right|}{L_{i}}}.$$

By following similar steps to the repulsive kernel, we find


(2.10) $$q_{T}(x)=\frac{1}{L_{R}}q_{T_{R}}(x)-\frac{\alpha }{L_{A}}q_{T_{A}}(x),$$

where


(2.11) $$q_{T_{i}}(x)=\frac{1}{e^{\frac{2\pi }{L_{i}}}-1}sgn(x)\left[e^{\frac{2\pi -|x|}{L_{i}}}-e^{\frac{\left|x\right|}{L_{i}}}\right].$$

### 2.3. Solution in one dimension

Let us assume that our objective is to attain a chosen density $\bar{\rho }(x,t):{𝕊}^{1}\times {\mathbb{R}}_{+}\to {\mathbb{R}}_{+}$ for the swarm. To find the forcing term f necessary to obtain the desired density, we assume that $\bar{\rho }$ satisfies equation (2.1), such that


(2.12) $$(\bar{\rho }f{)}_{x}=-{\bar{\rho }}_{t}-(\bar{\rho }{\bar{v}}_{\rho }{)}_{x},$$

with ${\bar{v}}_{\rho }(x,t)=\int_{-\pi }^{\pi }q_{T}\;(x-y)\bar{\rho }(y,t)\;dy$ . By integrating equation (2.12), assuming $\bar{\rho }\neq 0$ for any x , we obtain


(2.13) $$f(x,t)=-{\bar{v}}_{\rho }(x,t)-\frac{1}{\bar{\rho }(x,t)}\int {\bar{\rho }}_{t}(y,t)\;dy+\frac{1}{\bar{\rho }(x,t)}A(t),$$

with A(t) being a function of time only. We note that A(t) may not be chosen freely, as f should satisfy the integral condition $\int_{-\pi }^{\pi }\;f(x,t)\;dx=\int_{-\pi }^{\pi }\;q_{T}(x)\;dx\;\int_{-\pi }^{\pi }\;\rho_{tr}(x,t)\;dx$ , according to Fubini’s theorem [46]. As typically we consider odd kernels $q_{T}$ , we must have that f integrates to zero over the domain. Thus, A(t) is chosen to instantaneously satisfy this condition.

Once f is computed in closed form, we can derive $\rho_{\mathrm{tr}}$ from the deconvolution of equation (2.3). Such an operation depends on the interaction kernel $q_{T}$ . For standard interactions kernels introduced in §2.2, we have established a closed-form analytical solution.

**Repulsive kernel.** For the repulsive kernel [47], we can expand the convolution in equation (2.3) as


(2.14) $$\begin{array}{l} \begin{array}{rl} f(x,t) & =\frac{1}{e^{\frac{2\pi }{L}}-1}[e^{\frac{2\pi -x}{L}}\int_{-\pi }^{x}e^{\frac{y}{L}}\;\rho_{tr}(y,t)\;dy\\ & -e^{\frac{x}{L}}\int_{-\pi }^{x}e^{-\frac{y}{L}}\;\rho_{tr}(y,t)\;dy\\ & +e^{\frac{2\pi +x}{L}}\int_{\pi }^{x}e^{-\frac{y}{L}}\;\rho_{tr}(y,t)\;dy\\ & -e^{-\frac{x}{L}}\int_{\pi }^{x}e^{\frac{y}{L}}\;\rho_{tr}(y,t)\;dy]. \end{array} \end{array}$$

By taking two derivatives with respect to x , we find


(2.15) $$f_{xx}=\frac{f}{L^{2}}+2(\rho_{tr}{)}_{x}.$$

Thus, we can retrieve by integration


(2.16) $$\rho_{tr}\;(x,t)=\frac{1}{2}\int \left(f_{xx}-\frac{f}{L^{2}}\right)\;dx+B(t),$$

where B(t) is an arbitrary function of time.

**Morse kernel.** With the Morse kernel, we can write the convolution in equation (2.3) as


(2.17) $$f(x,t)=\frac{1}{L_{R}}f_{R}(x,t)-\frac{\alpha }{L_{A}}f_{A}(x,t),$$

where


(2.18) $$f_{i}(x,t)=\int_{-\pi }^{\pi }q_{T_{i}}(x-y)\rho_{tr}(y,t)\;dy.$$

From equation (2.16), we find


(2.19) $$\begin{array}{l} \begin{array}{rl} \rho_{tr}(x,t) & =\frac{1}{2}\int \left(f_{R_{xx}}-\frac{f_{R}}{L_{R}^{2}}\right)\;dx+C(t)\\ & =\frac{1}{2}\int \left(f_{A_{xx}}-\frac{f_{A}}{L_{A}^{2}}\right)\;dx+D(t). \end{array} \end{array}$$

However, from equation (2.13), we only have access to f , not to $f_{R}$ and $f_{A}$ . From differential and algebraic manipulations of equations (2.17) and (2.19), we find a problem in $f_{R}$ and f only:


(2.20) $$\begin{array}{l} \begin{array}{rl} \left(1-\frac{L_{A}}{\alpha L_{R}}\right) & f_{R_{xx}}-\frac{1}{L_{R}^{2}}\left(1-\frac{L_{R}}{\alpha L_{A}}\right)\;f_{R}\\ & =-\frac{L_{A}}{\alpha }\;\left(f_{xx}-\frac{f}{L_{A}^{2}}\right). \end{array} \end{array}$$

Equation (2.20) can finally be solved for $f_{R}$ through the use of spatial Fourier series.

The distribution of traces that generates the desired swarm density is not unique. In fact, the solution is defined up to an additive term ${\tilde{\rho }}_{\mathrm{tr}}(x,t)$ such that, for any x ,


(2.21) $$\bar{\rho }(x,t)\int_{-\pi }^{\pi }q_{T}(x-y){\tilde{\rho }}_{tr}(y,t)\;dy=B(t).$$

In particular, any static equilibrium $\rho_{\mathrm{eq}}(x)$ of equation (2.1), with corresponding velocity $v_{eq}(x)=\int_{-\pi }^{\pi }\;q_{T}(x-y)\rho_{eq}(y)\;dy=0$ for any x , is a solution of equation (2.21). For example, for a purely repulsive kernel with interaction length L and a static desired density, we obtain


(2.22) $$\rho_{tr}=-\bar{\rho }+B,$$

where B is an arbitrary constant.

### 2.4. Numerical method

To study the time-evolution of the equations of motion, we derive a numerical solution. Specifically, to solve the hyperbolic equation in equation (2.1), we rely on a finite volume method, which naturally accounts for conservation of a given quantity and for the finite velocity of perturbations [48]. We divide the domain [−π,π) in $N_{C}$ cells $C_{i}$ ( $i=1,\ldots ,N_{C}$ ) with uniform size Δx and consider as variables the average value of the density over the cells at time $t_{n}$ :


(2.23) $$Q_{i}^{n}=\frac{1}{\Delta x}\int_{C_{i}}\rho (y,t_{n})\;dy.$$

The evolution of this quantity in time is governed by


(2.24) $$Q_{i}^{n+1}=Q_{i}^{n}-\frac{\Delta t}{\Delta x}\left(F_{i+\frac{1}{2}}^{n}-F_{i-\frac{1}{2}}^{n}\right),$$

where Δt is the integration time-step and $F_{i+\frac{1}{2}}^{n}$ and $F_{i-\frac{1}{2}}^{n}$ are the numerical fluxes on the right and left interfaces, which approximate the value of the flux f(ρ)=ρv(ρ) at the interact over the time-step:


(2.25) $$F_{i-\frac{1}{2}}^{n}=\frac{1}{\Delta t}\int_{t_{n}}^{t_{n+1}}f(\rho (x_{i-\frac{1}{2}},t))\;dt.$$

For simplicity, we consider the Lax–Friedrichs method, which reads


(2.26) $$F_{i-\frac{1}{2}}^{n}(Q_{i-1}^{n},Q_{i}^{n})\approx \frac{1}{2}\left[\;f(Q_{i-1}^{n})+f(Q_{i}^{n})\right]-\frac{\Delta x}{2\Delta t}(Q_{i}^{n}-Q_{i-1}^{n}).$$

In 1D simulations, we utilize $N_{C}=500$ cells and a time-step of Δt=0.1 . To quantify the error between actual density of the swarm and desired one, we leverage the Kullback–Leibler (KL) divergence of the actual density of the swarm from the desired one over time, which represents a form of statistical distance [49].

### 2.5. Discrete framework

All the results in the previous subsections are only valid at the continuum-level description of the swarm. To validate our overall approach in more realistic conditions, we conduct 1D simulations of the discrete swarm. We consider the problem of coordinating N units of a swarm, each with mass m=M/N . The dynamics of the units is described by their position $x_{i}(t)$ along the circle, and is governed by a discretized version of equations (2.1), (2.2), and (2.3), namely [43],


(2.27) $${\dot{x}}_{i}=m\sum_{j\neq i}q_{T}\;(x_{i}-x_{j})+f_{i},$$

with $f_{i}$ being the external forcing on $x_{i}$ . This term is provided by the interaction of the units with the $N_{\mathrm{tr}}$ discrete traces at positions $x_{i}^{\mathrm{tr}}$ :


(2.28) $$f_{i}=m\sum_{k=1}^{N_{tr}}q_{T}\left(x_{i}-x_{k}^{tr}\right).$$

The desired configuration of the swarm is imposed in terms of density, so that an initial continuification is not required. Once the desired density of the traces is computed, self-equilibrating solutions are added to ensure that the density is positive everywhere. The resulting distribution of traces is discretized over a certain, fixed number of cells, such that traces can be added to the simulation. The number $N_{\mathrm{tr}}$ of traces is a result of the algorithm, as we approximate it as the closest integer to $\left(\int_{-\pi }^{\pi }\;\rho_{tr}\;dx\right)/m$ . Upon simulating the dynamics of the discrete system, we can define the corresponding continuum counterpart through continuification, which is achieved from kernel density estimation with a manually tuned Gaussian estimation kernel [50]. In this case, the KL divergence is evaluated on the continuified distribution.

### 2.6. Extension to multi-dimensional problems

Our method can be readily extended to multi-dimensional problems. Specifically, we consider a density $\rho :{𝕊}^{n}\times {\mathbb{R}}_{+}\to {\mathbb{R}}_{+}$ , where n=2 or n=3 and ${𝕊}^{n}$ is the n -sphere parametrized with $[-\pi ,\pi {)}^{n}$ , and extend equation (2.1) to


(2.29) $$\rho_{t}+\nabla \cdot (\rho v)=0,$$

with $𝐯:{𝕊}^{n}\times {\mathbb{R}}_{+}\to {\mathbb{R}}^{n}$ being the velocity vector and ∇⋅(⋅) the n -dimensional divergence operator. Equation (2.2) is generalized as a multi-dimensional circular convolution with vectorial interaction kernel ${𝐪}_{T}:{𝕊}^{n}\to {\mathbb{R}}^{n}$ and external forcing term $𝐟:{𝕊}^{n}\times {\mathbb{R}}_{+}\to {\mathbb{R}}^{n}$ :


(2.30) $$v(x,t)=\int_{S^{n}}q_{T}\;\left(x-\tilde{x}\right)\rho \;(\tilde{x},t)\;d\tilde{x}+f(x,t).$$

The formula for 𝐟 is a multi-dimensional version of the circular convolution in equation (2.3), with a density of traces $\rho_{\mathrm{tr}}:{𝕊}^{n}\times {\mathbb{R}}_{+}\to \mathbb{R}$ .

In the multi-dimensional case, closed-form solutions to the problem are not available. Specifically, closed-form solutions to the periodicization of the interaction kernels in all spatial directions cannot be easily found. As such, we truncate the multi-dimensional infinite series extending equation (2.4) to a finite number of terms. For example, for n=2 ,


(2.31) $$q_{T}(x,y)\approx \sum_{k_{x},k_{y}=-K_{q}}^{K_{q}}\;q(x+2k_{x}\pi ,y+2k_{y}\pi ),$$

where 𝐪(x,y) is a non-periodic interaction kernel in 2D.

To obtain $\rho_{\mathrm{tr}}$ , we numerically solve an inverse problem for equation (2.29), substituting the density ρ of the swarm with the desired density $\bar{\rho }$ . In our 2D simulations, we utilize a finite volume approximation of the equation with a complete 2D Lax–Friedrichs flux. We utilize 30 cells to discretize each direction, for a total of 900 cells, along with a time-step of Δt=0.1 .

## 3. Results

We first demonstrate our approach on 1D problems over a ring, for which we propose a detailed analysis of the density of traces required to achieve a desired density of the swarm, along with the error over time (§3.1). Further, we validate our approach by simulating the same 1D problems with discrete swarms and traces. Then, in §3.2, we consider a 2D example of a complex, evolving formation problem of a swarm that can be achieved with our stigmergic approach.

### 3.1. Results in one dimension

We consider three applications of our approach in 1D: (i) a swarm that reaches a stationary formation; (ii) a swarm that forms a travelling wave in a non-dispersive medium, where the group and phase velocities are equal; and (iii) a swarm that forms a travelling wave in a dispersive medium, where the group and phase velocities are different.

As the waveform, we consider a von Mises function with mean μ and concentration coefficient k [51]:


(3.1) $$\bar{\rho }(x)=M\frac{1}{2\pi J_{0}(k)}e^{k\cos(x-\mu )},$$

with $J_{0}$ being the modified Bessel function of the first kind of order zero. In the case of a travelling wave in a non-dispersive medium, we let the function travel at a constant speed v , without affecting the shape of the wave, such that the mean varies with time as μ+vt . Finally, for the simulation of a travelling wave in a dispersive medium, we let $k(t)=k_{0}+A\;\sin(\omega t)$ to capture oscillations around a fixed value $k_{0}$ , with amplitude A and radian frequency ω .

**Figure 2.**
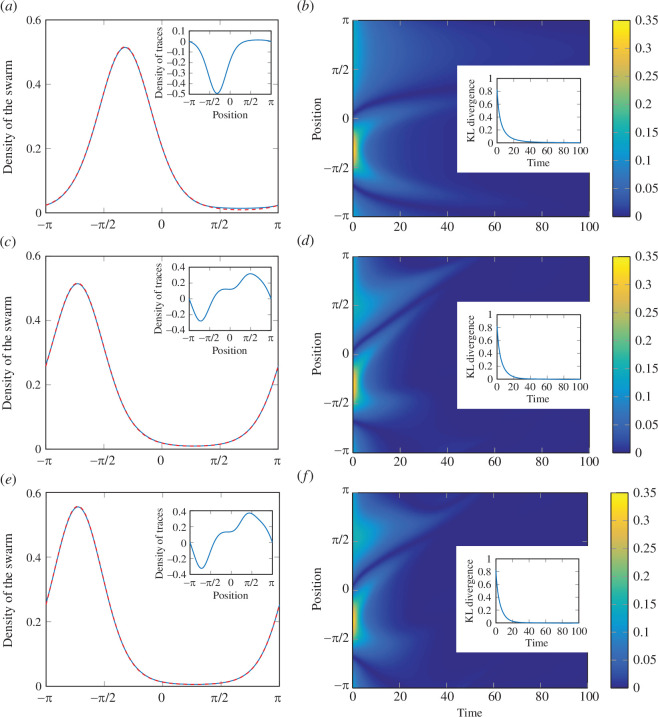
Results of one-dimensional, continuum-level simulations. (*a,c,e*) The profiles of the density of the swarm at the end of the simulation ( t=100 , blue solid line), along with the instantaneous desired profile of the density of the swarm (red dashed line), for the cases of stationary formation (*a*), travelling wave in a non-dispersive medium (*c*), and travelling wave in a dispersive medium (*e*); insets indicate the corresponding density of the traces. (*b,d,f*) The absolute error at each point in the spatial domain over time, for the cases of stationary formation (*b*), travelling wave in a non-dispersive medium (*d*), and travelling wave in a dispersive medium (*f*); insets indicate the KL divergence of the actual density of the swarm from the desired one over time.

We run a simulation for each of the three applications, utilizing a finite volume method to evolve the density of the swarm over time, as described in §2.4. All simulations start from a uniform density distribution with unitary mass. For all simulations, we consider a repulsive kernel with interaction length L=1 , set μ=−1 , and run the simulation until $t_{F}=100$ . For the stationary formation and the travelling wave in a non-dispersive medium, we fix k=2 . In both travelling wave simulations, we set v=0.05 . For the case of the travelling wave in a dispersive medium, we choose $k_{0}=2$ , ω=0.1 and A=0.5 , with an initial phase of −π . Results of these simulations are shown in figure 2, where we also consider the absolute error between actual density of the swarm and desired one and the KL divergence of the actual density of the swarm from the desired one over time. Videos of the simulations are available as electronic supplementary material.

In the case of stationary formation (figure 2*a*), the swarm is able to almost perfectly achieve the desired density ( KL<0.003 at the end of the simulation). As one may intuitively guess for a repulsive potential, traces have a higher density where the density of the swarm ought to be small. In particular, we find that the profile of the density of the traces closely resembles the opposite of the desired density of the swarm, see equation (2.16). Notably, we observe that there are areas where the density of traces is negative. The indeterminacy in the distribution of traces allows us to obtain a non-negative density. As clear from equation (2.16), the actual density of the traces can always be made positive from the addition of a constant (that is, a self-equilibrating field of traces). From the analysis of the error over time (figure 2*b*), we find that the error at the peak quickly decays to zero (more than 90% of its initial value within 10 time units), while the error in the tail of the function has a longer decay time (with similar decays in 50 time units).

Also when considering the travelling wave in a non-dispersive medium (figure 2*c*), we observe an almost perfect tracking of the desired formation ( $KL<10^{-5}$ after 60 time units). In this case, the density of the traces does not resemble the opposite profile as the instantaneous desired density, as it is corrected to anticipate the change in the desired density over time. Interestingly, we find that the KL divergence decays slightly faster than for the stationary formation, reaching KL<0.1 in 12.4 time units against 14.4 time units for the static case (figure 2*d*). This result may be explained by the faster decay in the error of the tail that follows the peak (initially on its left). This analysis is corroborated by the error over time, wherein one of the tails decays faster to zero error ( 90% decay from its initial value in about 20 time units) compared with the stationary formation in figure 2*b*, while the other translates at a velocity dictated by v .

For the travelling wave in a dispersive medium (figure 2*e*), we record similar profiles for the density of the swarm and traces as for the non-dispersive medium. The main difference is in the width of the peak of the distribution, which is wider for the dispersive case due to the modulation and the initial different phase. Despite the additional modulation of the waveform, we still obtain similar profiles for the error (figure 2*f*).

**Figure 3.**
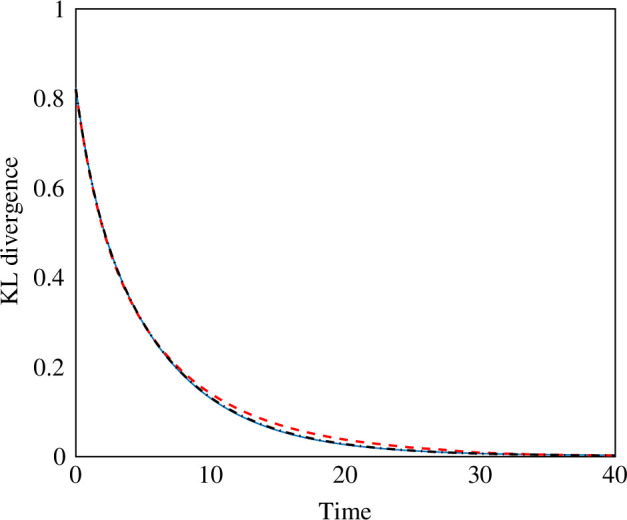
Results of 1D, discrete-level simulations. Blue solid, red dashed and black dash-dotted lines show the KL divergence of the actual density of the swarm from the desired one over time for the three cases of stationary formation, travelling wave in a non-dispersive medium, and travelling wave in a dispersive medium, respectively.

In addition to these simulations at a continuum level, we conduct analogous analyses with discrete swarms and traces (see §2.5). In particular, we replicate the three 1D studies at a discrete level. We consider very large swarms, with 5000 units, which would be complex to control with traditional strategies [52]. In figure 3, we show the KL divergence for the discrete simulations, computed from the continuified density of the discrete swarm: the three discrete swarms can all track their desired distribution. These simulations confirm the applicability of our approach in real, discrete swarms.

### 3.2. Results in two dimensions

**Figure 4.**
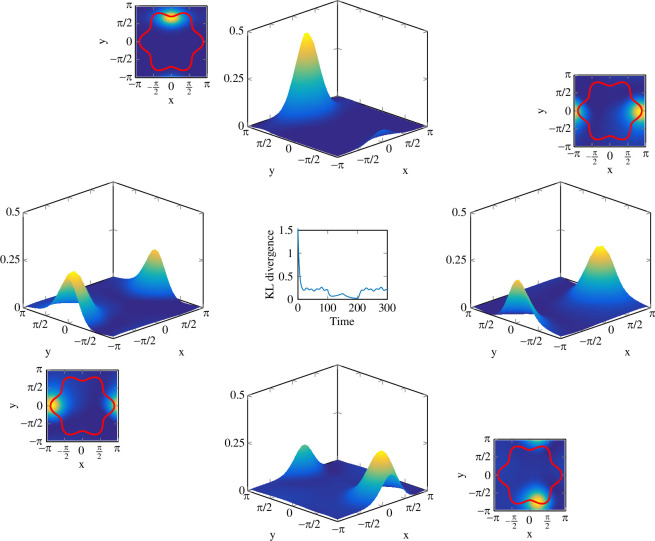
Results of 2D, continuum-level simulations. The figure shows a few snapshots of the density of the swarm, after a few time-steps ( t=1 , bottom), halfway through the first half of the wavy circle (right), as the breast of the lion is completely open (top), and halfway through the second half of the wavy circle (left). We show both 3D and top views. In the top view, the red circle indicates the desired trajectory of the centre of the swarm. The plot at the centre represents the KL divergence of the actual density of the swarm from the desired one over time.

Our approach can be easily extended to 2D and three-dimensional (3D) problems, as described in §2.6. Herein, we consider 2D simulations to show realistic applications of our method. Specifically, we consider the problem of a swarm inspired by Leonardo da Vinci’s lion robot [42]. This ‘automaton’ was designed by Leonardo to autonomously move on a trajectory, stop in front of Francis I King of France, opening its breast full of flowers, and return to its initial position. We use these steps as inspiration for a model example of coordination in 2D, where a swarm tracks and deforms over a wavy circle, similar to a flower with six lobes; see electronic supplementary material and figure 4. Specifically, we task the swarm to aggregate in a compact formation (figure 4, bottom) and reach a target location by moving along the wavy circle (figure 4, right). Upon reaching the target location, the swarm shall change the formation (simulating the lion opening its breast; figure 4, top) to perform a collective task (for example, collective construction). Upon completion, the swarm shall resume its original formation (representing the lion closing its breast) and return to the starting location by moving on the wavy circle (figure 4, left). Videos of the simulation are in the electronic supplementary material.

In these simulations, for simplicity, we assume quasi-static changes for the desired density, whereby we assume ${\bar{\rho }}_{t}=0$ in our computations. Similar to the 1D case, we start from a uniform distribution with unitary integral. We set the final time to $t_{F}=300$ , and divide this time-span in three phases with equal duration: translation along the first half of the wavy circle ( 0−100 ), opening/closing of the breast ( 100−200 ) and return to the initial position along the second half of the wavy circle ( 200−300 ). We require the swarm to rigidly translate along the trajectory while maintaining a formation described by a 2D von Mises function with $k_{x}=k_{y}=2$ . To design the desired trajectory, we let the mean of the 2D von Mises function translate along the wavy circle. The opening and closing of the lion’s breast is simulated by dynamically varying $k_{x}$ of the 2D von Mises function from 2 to 3 .

Our results indicate that our approach is successful in replicating the motion of Leonardo’s automaton (figure 4). Based on the KL divergence (figure 4, centre), the density of the swarm quickly reaches the desired one, with overall small differences ( KL<0.3 after 11 time units). After the initial settling, the KL divergence is larger in the first and third phases (around 0.25 ), while it decreases in the second phase during the opening and closing of the breast (around 0.10 ). In principle, adopting a non-quasi-static approach can decrease the KL divergence, paying the price of a higher computational cost.

## 4. Discussion and conclusions

Complex systems composed of many units often display surprising self-organization phenomena [53]. Self-organization is inevitably associated with information transfer between the units of the system, which occurs through direct social interactions or indirect cues mediated by the environment [54]. Stigmergy encapsulates the ability of the units of a system of transferring information among them by modifying the surrounding environment [1]. This concept has found a broad range of application in animal [2] and robotic [18] swarms, mixed societies of animals and robots [26], and even human social systems [55].

A particularly challenging endeavour in biology is the analysis of how stigmergic signals left in the environment lead to self-organization and execution of complex tasks, even in the absence of central coordination. Such a challenge persists in engineering design of stigmergic modifications of the environment in order for the swarm to behave as desired. This problem requires the formulation of mathematically tractable and interpretable models to be used in the analysis of swarms and the design of control systems. However, literature on stigmergy typically focuses on agent-based models, where each unit of the swarm follows a set of behavioural rules. These models are difficult to use for holistic analyses of environmental modifications and control purposes.

Here, we propose a new mathematical framework to study stigmergy in swarms. Through this framework, we lay out how traces should be left in the environment to enable the coordination of a swarm, provided a mathematical model for the motion of the swarm is available. Such a framework is rooted in recent ideas for the control of large multi-agent systems [30,31], which draw on the analogy between robots and particles in a fluid. While for small groups of particles we can describe the motion of each individual unit, when considering large systems a continuum description is preferable. Thus, we continuify the problem by modelling the swarm as a fluid, design the control at a continuum level in terms of density of traces, and discretize this distribution to deploy them in the environment. This approach transforms the control problem on a large number of coupled ordinary differential equations to that on a single partial differential equation, which becomes analytically tractable.

One of the potential applications of the proposed setting is collective construction in robotic swarms through collective AM, where buildings and infrastructure are built through layer-by-layer deposition [56–59] by a team of robots [60]. This paradigm has been inspired by other efforts in collective construction with teams of robots using pre-made parts [61,62], amorphous deposition [63], or the robots themselves [64]. While initial efforts focused on ground-based robots [65–67], the recent breakthrough in Zhang *et al.* [68] demonstrated the possibility of aerial AM for large-scale buildings and infrastructure, going far beyond previous efforts that only focused on structure assembly through drones [69]. Robots in construction teams ought to reach a specific location and coordinate with each other. Our work provides a pathway to achieve these goals, making a first step toward collective AM algorithms.

Within the context of collective AM, traces may be left in the environment by the swarm or by one or more exogenous robots, not directly involved in the construction. While stigmergic cues could be provided by the built structure itself [22,23], it is tenable that in many applications the trace deposition process should be independent from the printing. A potential implementation of the trace deposition entails the use of a separate nozzle, utilizing a coloured dissoluble material that can be identified by other robots. Traces can be left as signs on printed structures or as thin marks on the ground and removed after construction, similar to supporting material used in desktop 3D printers [70]. Beyond collective AM, the traces needed for our algorithm may assume different forms. For example, they may represent actual fixed, physical objects, an additional swarm of mobile robots, spatially extended continuous robots that can actively deform or other environmental modifications that affect the swarm (such as changes in background lighting in light-foraging swarms [71]).

Regardless of the application, we assume that units in the swarm are not able to distinguish between other units and traces. Such a possibility is in practice verified in large swarms where individual units have limited computing power and may only perform simple operations to avoid collisions with surrounding entities, be they other units or traces. When a unit can discern traces from other units, we have an additional degree of freedom, as we can modify the type of interaction units have with traces. For example, should units be able to distinguish different types of traces, negative densities of traces may be implemented in practice by using traces encoding interactions via an attraction potential.

Interestingly, we find that the distribution of traces has several degrees of indeterminacy. Such indeterminacy is not a drawback, but rather an advantage of the method, as it provides additional degrees of freedom for the control and swarm designer. For example, if traces represent fixed, physical objects that cannot be removed or relocated after they have been released, their density can only increase over time at each point of the domain: the indeterminacy of the distribution of traces allows to satisfy this condition.

We demonstrate the validity of our framework in a variety of simulations in 1D and 2D. In 1D, we show that the swarm can achieve a stationary formation, and that we can make this stationary formation rigidly translate similar to a travelling wave in a non-dispersive medium. We validate our coordination strategy with even more complex scenarios, as we studied a concurrent modification of the formation during the translation of the wave, simulating a travelling wave in a dispersive medium, where different harmonics of the waves travel at a different speed, thus modifying the waveform over time. Analogous simulations at a discrete level demonstrated the validity and practical viability of our approach, even in very large-scale swarms with thousands of units.

In 2D, we selected a historical example to show the potential of our approach. Specifically, we replicated the behaviour of Leonardo da Vinci’s lion-shaped automaton, which was designed to pay homage to the King of France through its complex movements. We showed that our approach allows the swarm to perform composite, time-modulated tasks, tracking an uneven trajectory, stopping at a precise location, modifying the formation in time, and resuming its motion along the trajectory. All these behaviours can be easily achieved through a stigmergic approach.

Our approach does not come without limitations. In particular, we acknowledge that further work is needed to support real-world implementation. The lack of an explicit model for the trace deposition process benefits the generality of the theory, but it limits a prompt translation of the proposed approach to practice. Further, robustness of the approach to different forms of disturbances should be tested, and additional research is required for deployment in collective AM. Further, we acknowledge that the proposed control strategy is not suitable for all collective tasks, especially for small teams in which coordination and localization within an absolute frame are more easily achieved. Nevertheless, we believe that the proposed mathematical framework offers a powerful and adaptable tool to design environmental modifications for swarm coordination, leaving designers the versatility to implement them in different ways.

## Data Availability

All the data are included in the paper or in the code accessible at GitHub [72]. Electronic supplementary material is available online [73].
